# Loss of mGluR5 in D1 Receptor-Expressing Neurons Improves Stress Coping

**DOI:** 10.3390/ijms22157826

**Published:** 2021-07-22

**Authors:** Luca Zangrandi, Claudia Schmuckermair, Hussein Ghareh, Federico Castaldi, Regine Heilbronn, Gerald Zernig, Francesco Ferraguti, Arnau Ramos-Prats

**Affiliations:** 1 Department of Neurology, Charité—Universitätsmedizin Berlin, Corporate Member of Freie Universität Berlin and Humboldt-Universität zu Berlin, 10117 Berlin, Germany; luca.zangrandi@charite.de (L.Z.); regine.heilbronn@charite.de (R.H.); 2 Institute of Pharmacology, Medical University of Innsbruck, 6020 Innsbruck, Austria; claudia.schmuckermair@i-med.ac.at (C.S.); federico.castaldi@i-med.ac.at (F.C.); francesco.ferraguti@i-med.ac.at (F.F.); 3 Department of Psychiatry 1, Medical University of Innsbruck, 6020 Innsbruck, Austria; hussein.ghareh@i-med.ac.at (H.G.); gerald.zernig@i-med.ac.at (G.Z.)

**Keywords:** mGluR5, D1, stress, anxiety, memory, social behavior

## Abstract

The metabotropic glutamate receptor type 5 (mGluR5) has been proposed to play a crucial role in the selection and regulation of cognitive, affective, and emotional behaviors. However, the mechanisms by which these receptors mediate these effects remain largely unexplored. Here, we studied the role of mGluR5 located in D1 receptor-expressing (D1) neurons in the manifestation of different behavioral expressions. Mice with conditional knockout (cKO) of mGluR5 in D1 neurons (mGluR5^D1^ cKO) and littermate controls displayed similar phenotypical profiles in relation to memory expression, anxiety, and social behaviors. However, mGluR5^D1^ cKO mice presented different coping mechanisms in response to acute escapable or inescapable stress. mGluR5^D1^ cKO mice adopted an enhanced active stress coping strategy upon exposure to escapable stress in the two-way active avoidance (TWA) task and a greater passive strategy upon exposure to inescapable stress in the forced swim test (FST). In summary, this work provides evidence for a functional integration of the dopaminergic and glutamatergic system to mediate control over internal states upon stress exposure and directly implicates D1 neurons and mGluR5 as crucial mediators of behavioral stress responses.

## 1. Introduction

The individual ability to control, contend, avoid, or escape a stressful experience has a critical impact on the well-being and survival of an organism [1]. Inability to select appropriate stress coping mechanisms leads to severe behavioral sequelae [2]. Thus, adopting an appropriate coping style upon acute or chronic, escapable or inescapable stress is critical for behavioral resilience to stress [3].

Previous studies have shown that exposure to acute [4] or chronic stress [5] can induce activation of the mesocorticolimbic dopaminergic (DA) system, and there is now accumulating evidence showing the importance of such circuitry in the modulation of stress-coping strategies [6,7,8]. One of the main target areas of DA projections arising from the ventral tegmental area (VTA) and substantia nigra (SN) is the nucleus accumbens (NAc) with most of VTA DA neurons projecting to the shell and SN DA neurons to the core [9]. The NAc neuronal population is largely composed (90–95%) of two types of GABAergic medium spiny neurons (MSN) that express either D1 or D2 receptors. Dysregulation of the balance between D1- and D2-positive MSN activity in the NAc and striatum is postulated as an underlying cause for stress-related disorders such as depression [10]. Using cell-type-specific analysis, Lobo and colleagues [11] demonstrated that following chronic social defeat stress, susceptible mice showed an increase in ΔFosB expression in D2-MSN in the NAc core, NAc shell and dorsal striatum. Resilient mice, however, showed significantly higher levels of ΔFosB in D1-MSN across all striatal regions [11]. In addition, ablation of NAc D1, but not D2 receptors, was shown to disrupt the normal stress-coping behaviors in animals exposed to inescapable stress [12]. Similarly, it has been shown that repeated restraint stress reduces the strength of excitatory synapses on D1-MSNs, but not on D2-MSNs of the NAc core, indicating that changes in excitatory neurotransmission on D1-MSN could account for the induction of anhedonia [13].

Altered glutamatergic receptor activity has been associated with the development of stress-related psychopathology [14]. Due to their spatially restricted distribution within the synapse and fine-tuning of synaptic events such as long-term potentiation (LTP) and long-term depression (LTD), metabotropic glutamate receptors (mGluRs), rather than ionotropic glutamate receptors, are an ideal therapeutic target for many neurological disorders [15,16]. Among the eight members of the mGluR family, mGluR5 seems to have a pivotal role in stress-related disorders, such as anxiety, depression, and substance abuse [15,17,18,19]. Human studies exploring mGluR5 binding in patients suffering from anxiety, major depressive disorder, and post-traumatic stress disorder (PTSD) showed a close relationship between symptom severity and mGluR5 levels [20,21,22]. In addition, a vast number of preclinical studies indicated that antagonism at mGluR5 results in anxiolytic [23] and antidepressant [24] responses in experimental animals. Therefore, the effect of mGluR5 antagonism observed in preclinical studies might be the consequence of an increased resilience to stressful situations. Since adoption of dysfunctional stress coping strategies is a crucial non-genetic risk factor of anxiety and depression, pharmacological interventions targeting mGluR5 could have important clinical applications [19].

Despite the literature on the behavioral and physical consequences of acute and chronic inescapable stress being extensive, only a few studies have addressed the role of mGluR5 receptors in mediating adaptive coping strategies upon exposure to different types of stress. Germline mGluR5 knockout mice or mice receiving the mGluR5 antagonist MTEP showed maladaptive stress coping mechanisms, adopting active responses under inescapable stress exposure [25] and passive stress coping strategies under escapable stress [26]. Thus, although mGluR5 has been shown to be crucial in the selection of the behavioral strategies in response to stress, it remains largely unknown in which neuronal subtypes mGluR5 activity is required to modulate stress-related behavior. Given that the expression of mGluR5 and D1 overlap in brain regions important for stress processing, such as the NAc [27], striatum [28], prefrontal cortex [29], and amygdala [30], we hypothesized that mGluR5 in D1-expressing neurons are crucial mediators of the behavioral coping strategy elicited by acute stress exposure.

In this study, we report that the conditional knockout of mGluR5 in D1-expressing neurons elicits enhanced adaptive coping strategies in response to acute stress, while leaving intact motor abilities and behavioral domains related to memory, social behaviors, and anxiety.

## 2. Results

### 2.1. mGluR5 cKO in D1 Neurons Affects Stress Coping

**Figure 1:**
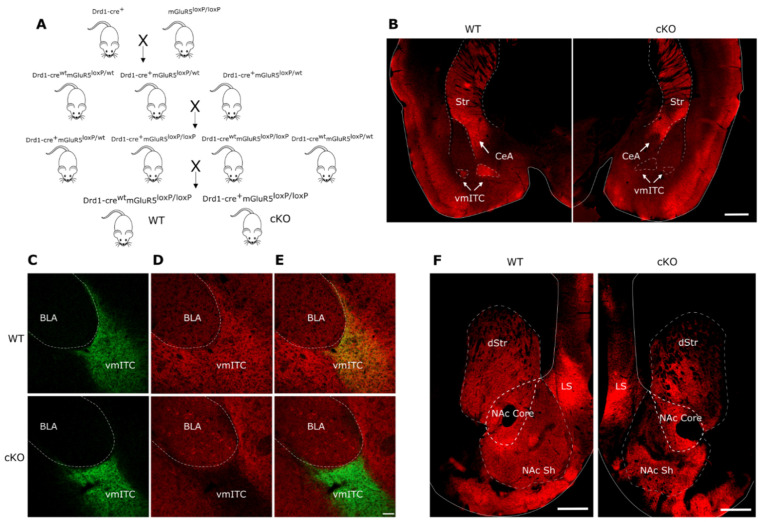
(**A**) Breeding strategy for the generation of mGluR5^D1^ WT and cKO mice. (**B**) Example image of mGluR5 expression in the cortex, striatum, and amygdala of mGluR5^D1^ WT (left panel) and cKO mice (right panel). Scale bar: 500 μm. (**C**) Example image of D1 receptor expression (green) in the vmITC of mGluR5^D1^ WT (top panels) and cKO mice (bottom panels), (**D**) mGluR5 expression (red), and (**E**) overlapped images. Scale bar: 40 μm. (**F**) Example image of mGluR5 expression throughout the striatum and NAc of mGluR5^D1^ WT (left panel) and cKO mice (right panel). Scale bar: 500 μm. Area abbreviations: dorsal striatum (dStr), striatum (Str), lateral septum (LS), nucleus accumbens core (NAc core) and shell (NAc Sh), central amygdala (CeA), main nucleus of intercalated cells of the amygdala (vmITC), and basolateral amygdala (BLA).

To explore the role of mGluR5 in D1 neurons in modulating stress reactivity, we generated mice with a selective knockout of mGluR5 by crossing mGluR5^loxP/loxP^ mice [31] with a Drd1a-cre driver line expressing Cre recombinase only in a subset of olfactory, striatal and amygdalar D1 neurons [32,33] (Figure 1A). Effectiveness of conditional deletion (cKO) of mGluR5 by Cre-mediated recombination in the mGluR5^loxP/loxP^ line has been previously characterized [31,34,35]. To demonstrate the cKO of mGluR5 from D1 neurons, we performed immunoblots on protein extracts from brain areas with high macroscopic co-localization of mGluR5 and D1, namely the olfactory bulb and striatum, including both the ventral and dorsal components. In these areas, Cre is expressed by the vast majority of D1 neurons in the olfactory bulb, but only in a subpopulation of the ventral and dorsal striatum (see methods). A marked reduction of mGluR5 was detected in olfactory bulb tissue homogenates of the cKO mice, whereas only a minor reduction of overall mGluR5 expression was observed in the ventral/dorsal striatum, although this did not reach statistical significance (Supplementary Figure S1). The hippocampal formation was used as negative control, and as expected, mGluR5 levels were highly similar. We further investigated the deletion of mGluR5 in these mice using immunostaining of brain slices. An extensive lack of mGluR5 immunoreactivity in mGluR5^D1^ cKO mice, compared to littermate controls not expressing Cre recombinase (mGluR5^D1^ WT), was observed in the central nucleus of the amygdala (CeA) and the main cluster of the intercalated cell masses of the amygdala (vmITC) (Figure 1B–E). The extent of the knockout of mGluR5 in D1 neurons was less clearly visible in the NAc and was primarily restricted to the medial component of its shell (Figure 1F).

**Figure 2:**
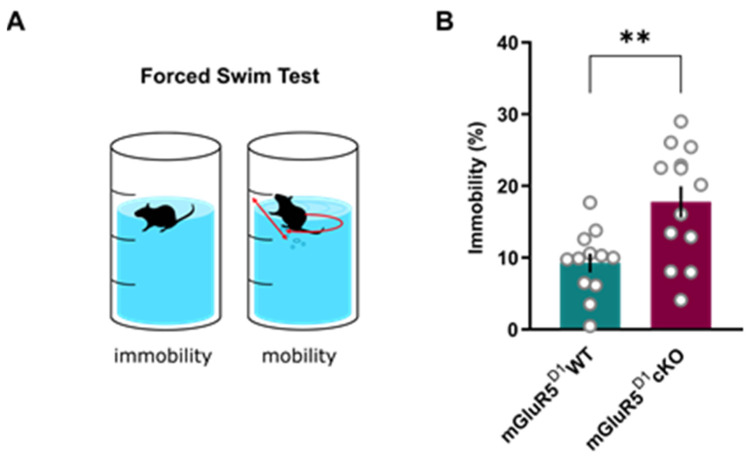
(**A**) Scheme of the forced swim test (FST). (**B**) Time spent immobile during the test (%). mGluR5^D1^ cKO mice showed higher time spent immobile than their WT littermates. Unpaired T-test, T = 3.49, *p* = 0.002. *n* = 12 mGluR5^D1^ WT, *n* = 13 mGluR5^D1^ cKO mice. ** *p* ≤ 0.01.

Given the strong co-localization of mGluR5 and D1 neurons in brain regions known to mediate stress coping mechanisms, such as the ventral striatum and amygdala, we tested the acute stress coping strategy in mGluR5^D1^ cKO mice using the FST (Figure 2A). In this test, mice are placed in a beaker filled with water and left undisturbed for 6 min, and while immobility constitutes an assessment of passive stress coping, mobility reflects active stress coping. In this test, mGluR5^D1^ cKO mice showed significantly more immobility compared to mGluR5^D1^ WT littermates (Figure 2B), thus reflecting a stronger adoption of a passive stress coping behavior than their WT littermates.

### 2.2. mGluR5 cKO in D1 Neurons Does Not Influence Baseline Anxiety

Previous studies have shown that mGluR5 antagonism and complete germline deletion of the mGluR5 affect learning and memory, social behaviors, and anxiety [31,36,37,38,39]. Negative allosteric modulation of mGluR5 induces potent anxiolytic actions in preclinical models as well as in humans [19]. However, recent findings suggest that mGluR5 might exert region- and cell-type-specific effects on anxiety [37,40,41,42] and thus, we sought to explore whether mGluR5 located at D1 neurons could mediate these behavioral expressions at baseline and explain the passive stress response of mGluR5^D1^ cKO mice in the FST.

**Figure 3:**
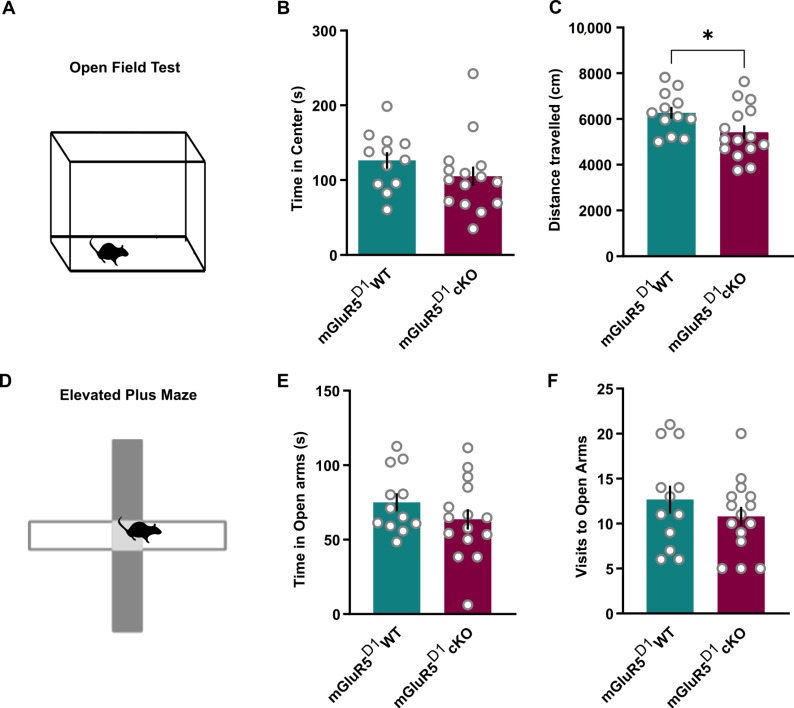
(**A**) Scheme of the open field test. (**B**) Time spent in the center of the open field (s). mGluR5^D1^ WT and cKO mice showed similar time spent in the center of the open field. Unpaired T-test, T = 1.20, ns (*p* = 0.24). (**C**) mGluR5^D1^ cKO mice travelled less distance during a 20 min test in an open field as compared to their WT littermates. Unpaired T-test, T = 2.086, *p* = 0.047. (**D**) Scheme of the elevated plus maze. (**E**) Time spent in the open arms of the maze (s). mGluR5^D1^ WT and cKO mice showed similar anxiety-like behavior as measured with time spent in the open arms: Unpaired T-test, T = 1.22, ns (*p* = 0.23). (**F**) Visits to the open arms of the maze. mGluR5^D1^ WT and cKO mice showed similar anxiety levels as measured with time spent in the open arms: Mann–Whitney test, U = 71.5, ns (*p* = 0.38). *n* = 12 mGluR5^D1^ WT, *n* = 15 mGluR5^D1^ cKO mice. * *p* ≤ 0.05.

When mGluR5^D1^ cKO mice were tested in the open field (Figure 3A), they displayed similar time spent in the center of the arena (Figure 3B) and travelled slightly less distance than WT mice (Figure 3C), suggesting that under anxiogenic conditions, these mice explore a novel environment less actively. An accelerating rotarod test to assess motor function excluded the possibility that these differences resulted from motor deficits in cKO mice (Supplementary Figure S2). To elucidate whether differences in exploratory behavior in the open field reflected anxiety-like measures, we tested these mice in the elevated plus maze (Figure 3D). In this test, mGluR5^D1^ cKO mice performed similarly to their WT littermates, exploring the open arms of the maze for a similar duration (Figure 3E) and with a similar frequency (Figure 3F), suggesting that mGluR5^D1^ cKO mice display normal anxiety-like behavior.

### 2.3. mGluR5 cKO in D1 Neurons Does Not Influence Memory or Social Behaviors

**Figure 4:**
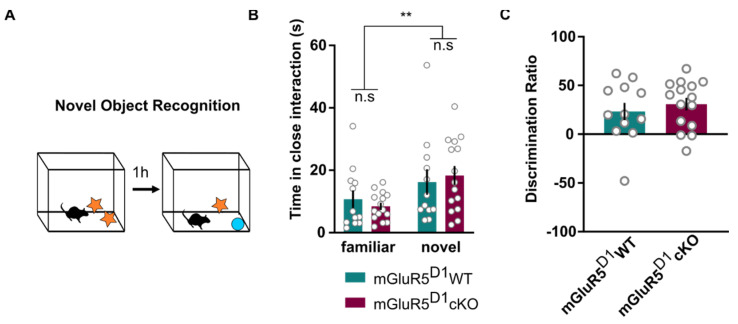
(**A**) Scheme of the novel object recognition behavioral paradigm. (**B**) mGluR5^D1^ WT and cKO mice showed similar recognition memory as measured with time spent interacting with the objects (s): Two-way ANOVA, main effect genotype: F(1, 25) = 0.001, ns (*p* = 0.97); main effect object: F(1, 25) = 11.41, *p* = 0.002; interaction effect: F(1, 25) = 0.94, ns (*p* = 0.34). (**C**) mGluR5^D1^ WT and cKO mice showed similar recognition memory, as measured with the discrimination ratio. Unpaired T-test, T = 0.69, ns (*p* = 0.49). *n* = 12 mGluR5^D1^ WT, *n* = 15 mGluR5^D1^ cKO mice. ** *p* ≤ 0.01.

Since baseline stress reactivity can strongly influence memory and social behaviors [43,44], we next assessed whether mGluR5^D1^ cKO and WT mice would differ in recognition memory, assessed using the novel object recognition test (Figure 4A). In this test, after a short familiarization with two identical objects, mice were allowed to explore the familiar object or a novel one an hour later. mGluR5^D1^ WT and mGluR5^D1^ cKO mice did not differ in measures of recognition memory in this test. Both mGluR5^D1^ WT and mGluR5^D1^ cKO mice spent more time in close interaction with the novel object in comparison to the familiar one (Figure 4B) and displayed similar novel object discrimination ratios (Figure 4C).

**Figure 5:**
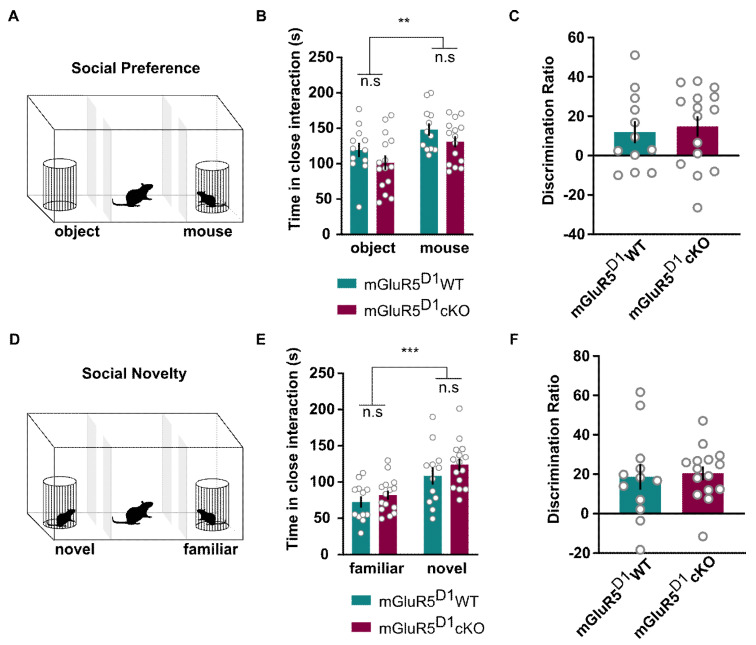
(**A**) Scheme of the social preference behavioral paradigm. (**B**) mGluR5^D1^ WT and cKO mice showed similar social preference as measured with time spent interacting with the object vs the mouse (s): Two-way ANOVA, main effect genotype: F(1, 25) = 3.28, ns (*p* = 0.08); main effect object/mouse: F(1, 25) = 10.86, *p* = 0.002; interaction effect: F(1, 25) = 0.003, ns (*p* = 0.96). (**C**) mGluR5^D1^ WT and cKO mice showed similar social preference, as measured with the discrimination ratio. Unpaired T-test, T = 0.38, ns (*p* = 0.70). (**D**) Scheme of the social novelty paradigm. (**E**) mGluR5^D1^ WT and cKO mice showed similar social novelty as measured with time spent interacting with the familiar vs the novel mouse (s): Two-way ANOVA, main effect genotype: F(1, 25) = 1.71, ns (*p* = 0.20); main effect novel/familiar: F(1, 25) = 27.73, *p* = 0.001; interaction effect: F(1, 25) = 0.16, ns (*p* = 0.69). (**F**) mGluR5^D1^ WT and cKO mice showed similar social novelty, as measured with the discrimination ratio. Unpaired T-test, T = 0.26, ns (*p* = 0.79); *n* = 12 mGluR5^D1^ WT, *n* = 15 mGluR5^D1^ cKO mice. ** *p* ≤ 0.01; *** *p* ≤ 0.001.

The impact mGluR5 function has on social behavior is thought to be crucially influenced by their location in specific brain circuits and different neuronal subtypes [35,37,39]. Given the importance of the NAc and amygdala in regulating social functioning [45,46], we thus sought to characterize social preference and novelty in mGluR5^D1^ cKO mice using a classical three-chamber approach [47] (Figure 5A,D).

During the social preference test, in which mice were allowed to freely explore a caged conspecific or an object (Figure 5A), mGluR5^D1^ cKO mice displayed normal social preference, as measured by longer time spent with the conspecific as compared to the object (Figure 5B), and similar social preference interaction ratios in comparison with mGluR5^D1^ WT mice (Figure 5C).

In the subsequent social novelty test, in which a novel interactor mouse is presented instead of the object, and test mice are allowed again to freely explore the apparatus (Figure 5D), mGluR5^D1^ cKO mice, like mGluR5^D1^ WT mice, spent more time exploring the novel conspecific as compared to the familiar one (Figure 5E) and showed similar discrimination ratios (Figure 5F). These data indicate that social and non-social interactions and recognition memory, regardless of their social or non-social nature, are not impaired in mice lacking the mGluR5 in D1 neurons.

### 2.4. mGluR5 cKO in D1 Neurons Enhances Adaptive Stress Coping Mechanisms

**Figure 6:**
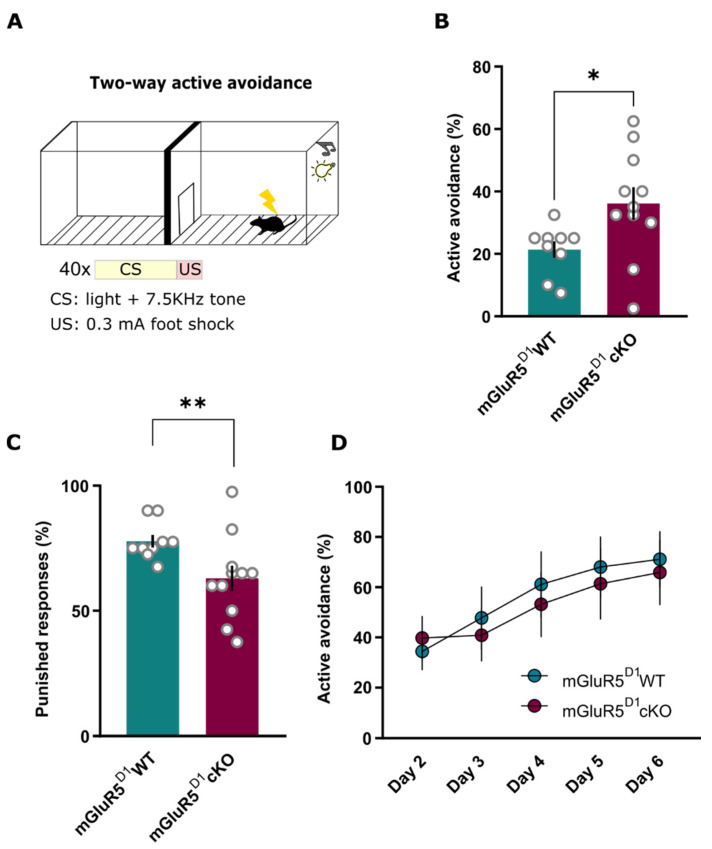
(**A**) Scheme of the two-way active avoidance (TWA) test. (**B**) mGluR5^D1^ cKO mice avoided more foot shocks during the first exposure (Day 1) to the TWA test than their WT littermates. Mann–Whitney, U = 18, *p* = 0.014. (**C**) mGluR5^D1^ cKO mice received less punished responses during the first exposure (Day 1) to the TWA test than their WT littermates. Mann–Whitney, U = 16.5, *p* = 0.010. (**D**) Both mGluR5^D1^ WT and cKO mice increased the number of avoided foot shocks with repeated testing in the TWA (Day 2–6) to a similar extent. Two-way ANOVA, main effect genotype: F(1, 25) = 0.081, ns (*p* = 0.77); main effect day: F(1, 25) = 8.97, *p* = 0.0001; interaction effect: F(1, 25) = 0.37, ns (*p* = 0.82); *n* = 9 mGluR5^D1^ WT, *n* = 11 mGluR5^D1^ cKO mice. * *p* ≤ 0.05; ** *p* ≤ 0.01.

Although the adoption of an active coping strategy in the FST is typically considered an antidepressant phenotype, increased time spent immobile does not necessarily imply a pro-depressive-like state, but more likely constitutes an adaptive stress response through a passive stress coping strategy towards an inescapable stressor [48]. To disentangle whether the passive strategy upon inescapable stress exposure resulting from the loss of mGluR5 in D1 neurons indeed represents an adaptive coping mechanism, we next addressed acute stress coping mechanisms in an escapable TWA task (Figure 6A). In this test, performed in a two-chamber apparatus, the presentation of a tone and a light (CS) predicts the delivery of a foot shock (US). The foot shock can be actively avoided or ceased when the animal shuffles to the opposite chamber during the CS presentation. Active avoidance, measured as successful shock avoidance, constitutes an assessment of active stress coping.

During the first test session day in the TWA paradigm, mGluR5^D1^ cKO mice actively avoided more shocks (Figure 6B) and were less punished (Figure 6C) in comparison to their WT littermates, thus suggesting an enhanced adaptive stress coping mechanism in response to acute stress. While the first exposure to the paradigm constitutes an assessment of the acute stress response, subsequent exposures reflect instrumental avoidance learning [49,50]. To assess whether mGluR5^D1^ cKO mice indeed displayed enhanced instrumental learning, we retested these mice in the same paradigm for the following 5 days. Both mGluR5^D1^ cKO mice and WT littermates steadily increased foot shock avoidances to a similar extent upon repetitive testing (Figure 6D), thus indicating that active stress coping strategies in these mice do not arise from enhanced instrumental avoidance learning.

Altogether, these data suggest that mGluR5^D1^ cKO mice display enhanced adaptive stress coping mechanisms, adopting passive strategies upon exposure to an inescapable stressor and active strategies upon exposure to escapable stress.

## 3. Discussion

Here, we show that mGluR5 expressed in a subset of D1 neurons of the NAc, striatum, and amygdala are crucial mediators of behavioral stress responses in mice. By phenotyping mice in which mGluR5 is selectively ablated in D1-expressing neurons in these areas, our work reveals the critical contribution of these receptors in D1 neurons in modulating adaptive stress coping strategies. Upon exposure to inescapable stress in the FST, mGluR5^D1^ cKO mice preferentially adopted a passive stress coping behavior while upon exposure to acute escapable stress in the TWA test, mGluR5^D1^ cKO mice adopted more active stress coping behavior when compared to their WT littermates. These effects did not seem to arise from differences in motor abilities, learning and memory, or different baseline anxiety-like behavior.

A large body of data has indicated that mGluR5 are extensively implicated in anxiety-like [37,51,52,53] and social behaviors [54,55,56,57,58]. However, their contribution to these and other behaviors seems to be highly dependent on their anatomical position in specific neuronal subpopulations and neural circuits. Moreover, the technique used to manipulate their activity in vivo (e.g., genetic vs pharmacologic) also appears to be crucial in defining the role these mGluR5 have in the modulation of these behaviors. For instance, while systemic antagonism of mGluR5 induces anxiolysis, germline deletion results in anxiogenesis [37,38]. Similarly, germline ablation of mGluR5 or selective ablation of the receptor in parvalbumin-positive neurons resulted in an apparent prosocial behavior [35,37], whereas its ablation from cortical principal cells did not produce any effect [59], thus depicting the complexity of the contribution of mGluR5 in emotional behaviors. In our study, we did not find any observable effect of mGluR5 cKO in D1 neurons on the expression of memory, anxiety-like or social behaviors.

The direction of mGluR5 modulation in mediating stress coping styles and stress resilience has been inconsistent across previous studies, reporting both resilience and susceptibility upon decreased mGluR5 function [25,26,53,60,61]. For example, whereas germline mGluR5 KO mice adopted an active coping stress style upon testing in the FST, which was suggested as an “antidepressant” response [25], several converging lines of evidence emerging from both exposure to escapable and inescapable stressors confirmed an endophenotype for stress-induced depression-like behavior in these mice [26]. These inconsistencies may arise from the exposure to inescapable stressors such as the FST and tail suspension test models, where the adaptive stress coping mechanism is a passive strategy [62] and is commonly misinterpreted as “depressed-like” [48]. Moreover, although substantial evidence supports the importance of glutamatergic modulation through mGluR5 in mediating stress responses [25,26,41,53,60,61,63], the position of these receptors in specific neuronal populations might differentially contribute to the observable behavioral effects [63].

Glutamatergic neurotransmission has long been known to affect the dopaminergic response to stress [64]. In particular, mGluR5 seem to closely interact with D1 receptors, converging in signal transduction pathways and in regulating striatal neurotransmission [28,65]. However, a general role for D1 neurons in stress coping mechanisms cannot be drawn from the extant literature. Preclinical studies elucidating the role of D1 neurons in the selection of stress coping styles and stress resilience suggest a dependence on the type of stressor and brain region in which they function [66,67,68]. For instance, while recent reports have shown that enhancing D1 neuron activity in the prefrontal cortex (PFC) and NAc results in resilient behavioral outcomes to stress [66,67], in other regions such as in the amygdala, increasing D1 activity results in stress-induced anxiogenesis [68]. Our study shows that mGluR5^D1^ cKO mice not only present an extensive ablation of mGluR5 in accumbal regions as previously described [27] but also in D1 neurons of the central nucleus of the amygdala and vmITC. Future studies are warranted to address whether the effects observed in stress coping of mGluR5^D1^ cKO mice result from a cumulative effect of these brain regions or are specifically mediated by one of them.

Studies in vitro using striatal cultures have shown that D1 and mGluR5 interact to activate ERK2 in a PKC-dependent manner and ultimately to modify downstream events, such as CREB phosphorylation [28]. To our knowledge, only a few in vivo studies have addressed signaling mechanisms or neurotransmission mediated by mGluR5 in D1-expressing neurons that could underlie their contribution in modulating stress coping. For instance, Fieblinger and colleagues demonstrated that D1R agonist-induced ERK1/2 phosphorylation in a mouse model of striatal denervation is mGluR5-dependent [69]. Similarly, García-Montes and colleagues found that downregulating mGluR5 in D1 neurons in a mouse model of Parkinson’s disease attenuated dyskinesia by decreasing ERK and FosB expression in the striatum [70]. In accordance with our findings of decreased novelty-seeking in the open field, previous studies have shown that mGluR5 knockdown in D1 neurons resulted in reduced novelty-seeking behavior and increased resilience to relapse to alcohol abuse and cocaine addiction in operant tasks [27,71]. Along the lines of these findings, a recent study has shown that specific deletion of mGluR5 in D1 neurons abolishes endocannabinoid (eCB)-mediated LTD in the NAc and prevents the expression of cue-induced reinstatement of drug-seeking behavior in mice [72]. An interesting open question is whether mGluR5 in D1-expressing neurons mediate stress-induced responses by influencing the eCB system. Given the tight relationship between stress and relapse to drug addiction, in which stress is often cited as a reason to relapse to drug use [73], these studies further support our findings describing the role of a specific subgroup of neurons expressing both D1 and mGluR5 as crucial mediators of behavioral stress coping.

## 4. Materials and Methods

### 4.1. Animals

All procedures involving animals were approved by the Austrian Animal Experimentation Ethics Board and were performed in compliance with the European Convention for the Protection of Vertebrate Animals used for Experimental and Other Scientific Purposes (ETS no. 123). Every effort was taken to minimize the number of animals used. mGluR5^loxP/loxP^ mice were crossed with Drd1a-Cre mice (Tg(Drd1a-cre)EY266Gsat (see Cre expression pattern details; Retrieved 11 July 2021, from: http://www.gensat.org/creGeneView.jsp?founder_id=33445&gene_id=48&backcrossed=false) to generate mice with deletion of mGluR5 specifically in D1 neurons. Since mGluR5 and D1 are highly expressed in the olfactory bulbs, and to avoid putative deficits in olfaction that might influence basic physiological functions, we assessed and confirmed that olfaction in cKO mice was unaffected (Supplementary Figure S2). To avoid possible deficits in maternal care, breeding was carried out using mGluR5^D1^ WT females and Cre positive (mGluR5^D1^ cKO) males. Animals were weaned at 4 weeks of age and group-housed in a climate-controlled facility on a 12 h/12 h light/dark cycle with lights on at 07:00 AM, with water and food ad libitum. Genotyping was performed from ear punches and determined by PCR. Only male mice aged 10-20 weeks old were used for behavioral experiments. All experiments were performed during the light cycle. Prior to all experiments, animals were handled for a minimum of two days and acclimatized to the testing rooms for behavioral phenotyping for at least 24 h. To reduce the number of animals used in this study, each mouse was tested in all paradigms described in the methods section in order of appearance, except for the stress-related and motor tests, for which independent cohorts of mice were used.

### 4.2. Buried Food Test

For this test, animals were food deprived overnight before testing. Mice were placed individually in a standard type 2 cage filled with 10 cm-deep bedding, where a food pellet was hidden, and left to explore. The latency to find the buried food with a maximum cutoff time of 10 min was manually annotated and used as an assessment of olfaction.

### 4.3. Accelerating Rotarod Test

Motor ability was assessed using the accelerating rotarod, using methods similar to those described in [74]. Briefly, mice were placed on the dowel with the rotarod rotating at 4 rpm, which was gradually accelerated to a maximum of 40 rpm with a 5 min test time cutoff. Each mouse underwent three trials per day, separated by a minimum of 30-s inter-trial interval, during two consecutive days. The latency to fall to the floor was automatically assessed by photocell beams and used as a measure of motor coordination and dexterousness.

### 4.4. Open Field and Novel Object Recognition Test

Open Field and Novel Object Recognition tests were performed in a sequential manner. On day 1, each mouse was individually placed in a squared open field arena (50 × 50 × 35 cm) made of grey opaque plastic and allowed to freely explore it for 20 min. Distance travelled (in cm) was taken as an assessment of locomotion and time spent in the center area (25 × 25 cm) as an approximation of anxiety-like behavior. Illumination was set at 30 Lux. On the following day, mice were placed in the same arena containing two identical objects for a 10 min familiarization trial. Object recognition memory was tested 1 h later during a 5 min discrimination trial in the arena containing a familiar and a novel object (a grey stone cylinder or a Lego block). Each trial was recorded with a video camera mounted on top of the arena and time spent investigating the objects (<5 cm from the object) was automatically scored using Ethovision XT 12 software (Noldus, Wageningen, the Netherlands; RRID:SCR_000441). The discrimination ratio was calculated as follows:

(Time spent investigating novel − time spent investigating the familiar object)/(total time investigating) × 100.

### 4.5. Social Preference and Novelty

Social behavior was assessed using a modified three-chambered social task apparatus [37]. The chamber was a rectangular box (75 cm long × 30 cm wide × 35 cm tall) made of an opaque glass and divided into three equal compartments, connected through rectangular doors (7 cm × 7 cm) allowing free exploration of each chamber. Mice were tested in the dark using infrared light (Lux < 5). The testing procedure involved two phases: social preference and social novelty. Each individual test mouse was placed in the center chamber and allowed to explore the entire apparatus for 10 min. Following this habituation period, mice were gently guided to the middle chamber by closing the side walls. Following this, a young (5–8 weeks) unfamiliar mouse was placed into a mesh container (15 cm tall, 7 cm diameter) in the middle of the least explored side chamber during the habituation phase, whereas an identical empty mesh container was placed in the middle of the opposite chamber. The test mouse was then allowed to explore the chambers for 10 min (social preference). For the second phase of the test, mice were again contained in the center chamber and another young unfamiliar mouse was placed in the chamber that previously contained the object (empty mesh cylinder), and test mice were allowed to freely explore the entire apparatus for 10 min (social novelty). Time spent in interaction with the mesh cylinders (<5 cm) was automatically tracked and scored using Ethovision XT 12 software (Noldus).

The social preference discrimination ratios were calculated as follows:

(Time spent investigating mouse − time spent investigating the object)/(total time investigating) × 100.

The social novelty discrimination ratios were calculated as follows:

(Time spent investigating a novel mouse − time spent investigating the familiar mouse)/(total time investigating) × 100.

### 4.6. Elevated Plus Maze

Mice were allowed to explore an elevated platform (72 cm above the floor) consisting of two opposing open (30 × 5 cm) and two opposing closed arms (30 × 5 cm) for a total of 10 min. Illumination in the open arms was set at 50 Lux. To start the test, mice were placed individually in one of the closed arms. The behavior of each mouse was tracked with Ethovision XT 12 software (Noldus). Arm entries were defined as the crossing of the center of mass of the animal. Measurements during the test included time spent in the open arms, and visits to the open arms. The position of the animal within the maze was automatically tracked and scored using Ethovision XT 12 software (Noldus).

### 4.7. Forced Swim Test

Mice were tested in a glass cylinder (15 cm diameter) filled with water (26–28 °C) in a room dimly illuminated (30 Lux). Each mouse was individually placed in the cylinder and left undisturbed for 6 min. The position of the animal within the glass cylinder and activity were automatically tracked and scored using Ethovision XT 12 software (Noldus) and individually validated by a trained experimenter blind to the genotype of the animals.

### 4.8. Two-Way Active Avoidance Test

The experimental procedure was performed as previously described [75]. Briefly, mice were tested using a fully automated setup (Ugo Basile, Gemonio, Italy), consisting of a two-chambered apparatus (47 × 18 × 26 cm) with equal sizes and an electrified grid floor. On the first test day, mice were individually placed in a randomly selected compartment and allowed to freely explore the apparatus for a 10 min habituation period. On subsequent testing days (day 2 to day 6), mice were habituated for 3 min prior to the test. Following the habituation period, a light and an 80 dB tone (7500 Hz) compound stimulus was presented for 15 sec, co-terminating in the last 5 s with a 0.3 mA foot shock, discontinued upon escape to the opposite chamber. Mice were exposed to a total of 40 trials/day. If the test mouse made a full transition to the opposite chamber during presentation of the compound stimulus, the foot shock delivery was avoided. Data collected from this test included percentage of avoided or punished trials.

### 4.9. Immunohistochemistry

For immunohistochemical characterization, mice were first deeply anaesthetized with thiopental sodium (150 mg/kg, i.p.) and transcardially perfused with a fixative (4% paraformaldehyde + 15% picric acid in 0.1 M phosphate-buffer (PB), pH 7.2–7.4). Following brain extraction, coronal sections were cut (50 μm) on a Leica VT1000S vibratome (Leica Microsystems, Vienna, Austria) and immunostained against mGluR5 and D1, based on previously described procedures [76]. A rabbit antibody against mGluR5 (Frontier Institute, Hokkaido, Japan; AB_2571802) and a goat antibody against D1 (Frontier Institute, AB_2571594) were diluted 1:1000 in 2% normal goat serum (NGS), 0.1% Triton X-100 in Tris-buffered saline (TBS; pH 7.4) and sections incubated for 48 h at 6 °C. Sections were then incubated overnight with the respective secondary antibodies (anti-goat Alexa Fluor™488, 1:1000, Jackson ImmunoResearch Europe Ltd.; anti-rabbit Cy3, 1:500, Invitrogen, ThermoFisher Scientific, Vienna, Austria). After three washing steps with TBS, sections were finally mounted onto gelatin-coated slides and coverslipped with Vectashield (Vector Laboratories, Burlingame, US). Immunofluorescent sections were examined using a Zeiss AxioImager M1 microscope or a confocal laser-scanning microscope (SP5, Zeiss, Oberkochen, Germany) for low and high-resolution image acquisition, respectively.

### 4.10. Western Blots

Mouse brain tissue (olfactory bulbs, striatum, and hippocampus) was extracted under a stereomicroscope and homogenized in ice-cold 25 mM Tris-HCl, pH 7.4, 50 µM phenylmethylsulphonyl fluoride, Pepstatin 1 µg/mL and complete EDTA-free protease inhibitors (Roche, Vienna, Austria) in a buffer containing 320 mM sucrose. The P2 fraction was obtained by sequential centrifugation at 1000 g and 17,000 g. Total proteins of cell lysate (20 μg/lane) were quantified by the Biuret assay (Carl Roth GmbH, Karlsruhe, Germany), denatured in Laemmli sample buffer containing 30 mM dithiothreitol (DTT) and heated for 5 min at 60 °C for SDS-PAGE on pre-cast Nupage 4–12% Bis-Tris gels (Invitrogen). Proteins (20 µg) were then electrophoretically transferred to polyvinylidene difluoride membranes (Hybond P; Amersham Biosciences, Little Chalfont, UK). Blots were blocked for 1 h in 5% dried skimmed milk in TBS-0.1% Tween 20 and incubated overnight at 4 °C with anti-mGluR5 (1:3000; Frontier Institute, AB_2571802) or anti-β actin (1:3000; cell signaling, Frankfurt am Main, Germany #3700) antibodies. Immunoreactive bands were detected by incubating the membranes in a horseradish peroxidase-conjugated secondary antibody (1:10000, Invitrogen) followed by the ECL Prime reagent. Chemiluminescence was visualized with the Fusion SL-4 Vilber Lourmat imaging system (Peqlab, Erlangen, Germany) and densitometric analysis was carried out using the ImageJ gel analyzer function.

### 4.11. Statistics

Data were analyzed with Prism 8 (GraphPad Software Inc., San Diego, US) software. Sample size was predetermined based on published studies, experimental pilots, and in-house expertise. Data are shown as mean + SEM with individual values plotted for each animal whenever applicable. Data distribution was tested for normality and analyzed accordingly with appropriate parametric or non-parametric statistical tests (see figure legends). Where applicable (see figure legends), significant effects following significant ANOVA were further analyzed using Bonferroni post hoc tests and *p* values less than 0.05 were considered statistically significant. * *p* ≤ 0.05; ** *p* ≤ 0.01; *** *p* ≤ 0.001.

## Data Availability

The data presented in this study are available on request from the corresponding author.

## Supplementary Materials

The following are available online at https://www.mdpi.com/article/10.3390/ijms22157826/s1.
