# Supplementary material for: Loss of mGluR5 in D1 Receptor-Expressing Neurons Improves Stress Coping
Source: Int J Mol Sci. 2021 Jul 22;22(15):7826. doi: 10.3390/ijms22157826 (PMC8346057; doi:10.3390/ijms22157826)
Supplement: Supplementary file 1 [file ijms-22-07826-s001.zip › ijms-1254189-supplementary.pdf]

Supplementary Data

Supplementary Figures

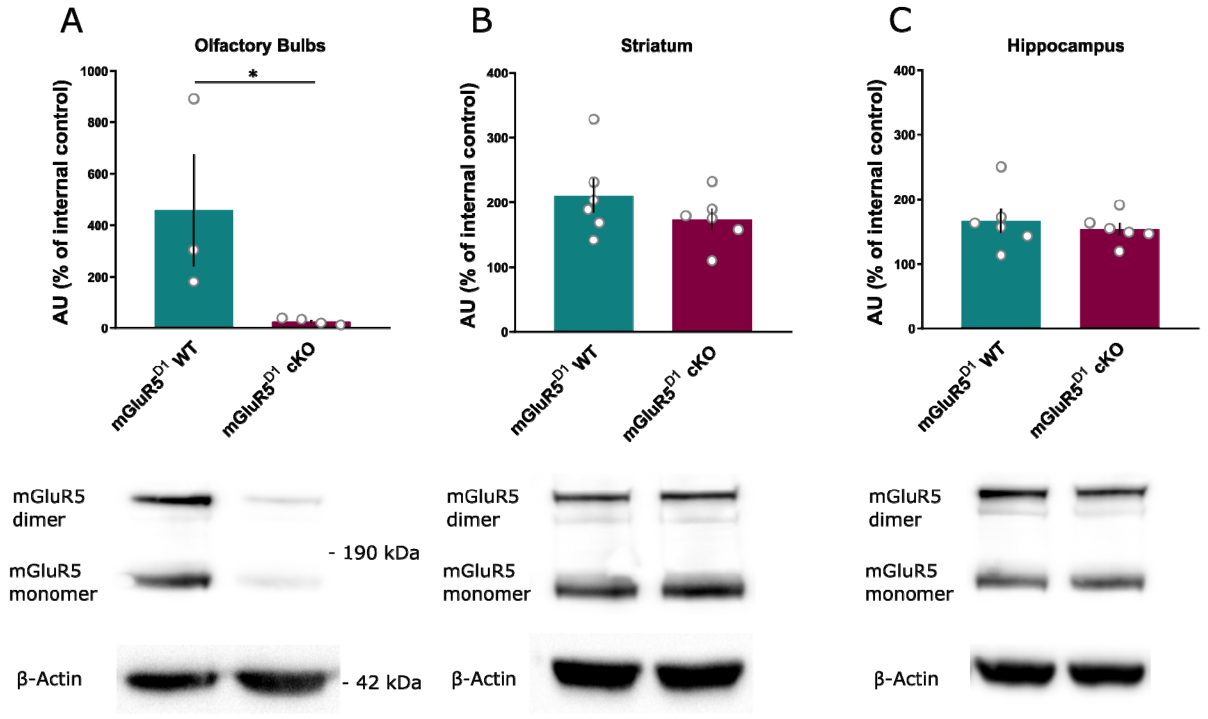

Figure S1. Relative mGluR5 protein level (arbitrary units, % of internal control) in WT and cKO mice (upper panels), with representative blot examples shown (lower panels), (**A**) in the olfactory bulb (Mann-Whitney; $U = 0$; $p = 0.028$; n= 3 mGluR5 WT, n=4 mGluR5 cKO), (**B**) striatum (Mann-Whitney; $U = 13$; $p = 0.24$, n= 6 mGluR5 WT, n=6 mGluR5 cKO) and (**C**) hippocampus (Mann-Whitney; $U = 16$; $p = 0.4$, n= 6 mGluR5 WT, n=6 mGluR5 cKO). Data are presented as individual values, mean ±SEM.

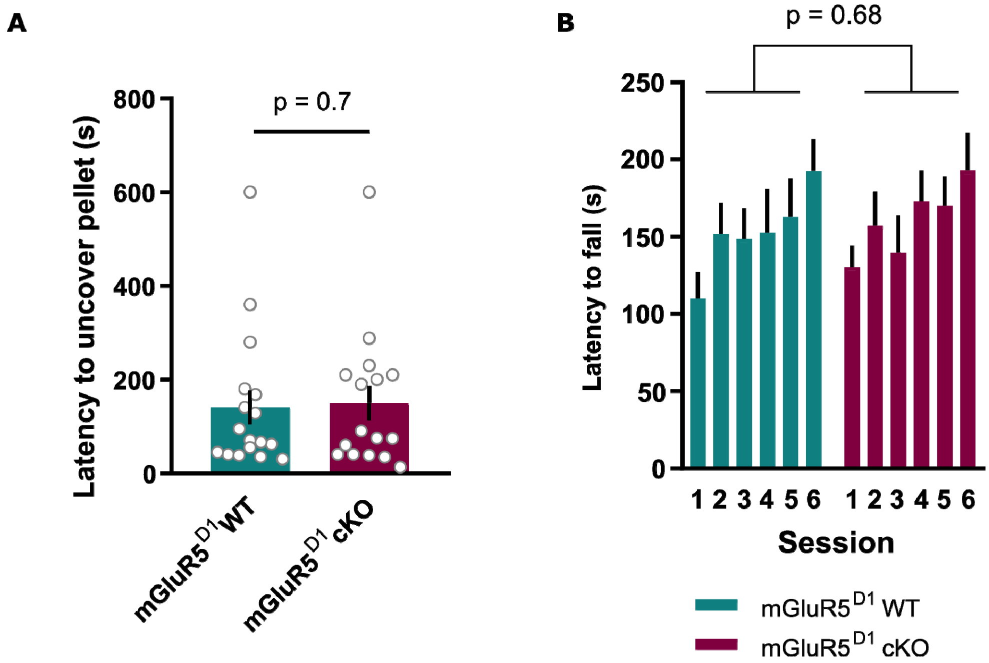

Figure S2. (**A**) The latency to uncover a food pellet (s) during a 10 min test was similar between mGluR5^{D1} WT (n=17) and cKO (n=16) mice (Mann-Whitney; U=127; p=0.75). Data are presented as individual values, mean ±SEM. (**B**) The latency to fall from an accelerating rotarod across six sessions was similar between mGluR5^{D1} WT (n=10) and cKO (n=10) mice: Two-way ANOVA, main effect genotype: F(1,18) = 0.176, ns (p = 0.68); main effect session: F(5,90) = 3.12, p = 0.012; interaction effect: F(5, 90) = 0.174, ns (p = 0.97). Data are presented as mean +SEM.
